# Your DNA, Your Say

**DOI:** 10.1080/20502877.2017.1314890

**Published:** 2017-05-18

**Authors:** Anna Middleton

**Affiliations:** ^a^ Society and Ethics Research, Connecting Science, Wellcome Genome Campus, Cambridge

**Keywords:** survey, genomics, film, attitudes, data sharing, GA4GH

## Abstract

Genomic and medical data sharing is pivotal if the promise of genomic medicine is to be fully realised. Social scientists working in the genomics arena ask the public ‘how is the technology working for you?’ Empirical studies on attitudes, values and beliefs are incredibly valuable; they offer a voice from those who are, or will be, directly affected. This is paramount if personalised medicine is to be truly *personal*. An International attitude study, Your DNA, Your Say, uses film to provide background information and an online survey to gather public views on donating one's own personal DNA and medical data for use by others. In this paper the rationale to the project is introduced together with an overview of the survey and film design. The project has been translated into multiple languages and the results will be used in policy for the Global Alliance for Genomics and Health.

## Introduction

### The relevance of genomic databases

Genomics is at the heart of personalised medicine (McDermott 2015). Using an individual's genetic profile to personalise a treatment or management pathway often starts with a DNA sequence, obtained from a sample of blood and/or tissue, e.g. from a tumour. Such sequencing is central to pioneering projects like the 100,000 Genomes Project in England (Genomics England 2017) and is now used as a first line test in some clinical services around the world (Takeichi *et al.* 2013).

When DNA is sequenced, bioinformatic pipelines are overlaid so that the data can be characterised, filtered, analysed and interpreted (Pabinger *et al.* 2014). One of the most significant steps in the interpretation process involves the comparison of a variant of interest with those contained in external databases. Such databases, e.g. Decipher (Chatzimichali *et al.* 2015) are helpful for exploring whether a particular DNA variant is likely to be indicative of a developmental disorder. Other databases, such as ExAC (Karczewski *et al.* 2017) offer clues about normal population variation and whether the sequence of interest has been commonly seen before. The key factor being that the human DNA sequence is so vast and has so much subtle variation between people that the only way to be sure of the significance of a particular variant is to assess how, and if, it has been catalogued previously.

Genomic databases are electronic catalogues, often online, populated with DNA code from a whole collection of people — varying ages, ethnicities, some healthy, some with disease. These databases may be publicly available or they may only be accessed with authorisation. The data contained may be de-identified, i.e. identifying information such as names and dates of birth have been removed, or they may have identifiers attached.

### Why genomic data sharing is necessary

Genomic data, that sit in databases, are shared with clinicians, non-profit researchers and for profit researchers every second, via the Internet, around the world. Medical doctors may use the databases to answer questions such as, ‘Is my patient's DNA sequence found in other people with the same disease?’ Non-profit researchers might explore, for example, ‘what's the prevalence of a particular genetic variant across Europe?’ and for-profit researchers might use the databases to answer, ‘is this particular medicine working in people with a specific type of DNA variation?’

The common denominator between the databases of human genomic information is that the DNA profile contained within, belonged prior to donation, to a real person. It is also clear that as genomics becomes firmly established in healthcare that the databases used in sequence interpretation need to be bigger and more heterogeneous than they are at present, so that they are much more representative of the full diversity of humankind.

The Global Alliance for Genomics and Health (GA4GH) is a non-profit organisation consisting of 450+ members from 40+ countries around the world (https://genomicsandhealth.org/members) all engaged in the data sharing endeavour. Its mission is:

to accelerate progress in human health by helping to establish a common framework of harmonized approaches to enable effective and responsible sharing of genomic and clinical data, and by catalyzing data sharing projects that drive and demonstrate the value of data sharing. (http://genomicsandhealth.org)

The GA4GH Members include both organisational and individual members covering non-profit research institutes, hospitals, biotech industry as well as for-profit research. One of the founding principles of the work of the GA4GH is that the donation of genomic and medical data by people, is fundamentally a ‘good thing’ and that this has to happen if personalised medicine is to be delivered effectively. This makes the assumption that the patient or research participant (i.e. people to whom the data originally belonged) are comfortable donating their data to start with. Early research is already showing that particular groups of patients with rare diseases accept the use of their data for use in certain types of research but not others (McCormack *et al.* 2016).

### Why are societal attitudes to genomic data sharing important?

Understanding societal attitudes towards genomic data donation and access by others is important because it is relevant to all of us. Genomic medicine is being ‘mainstreamed’ across whole healthcare services, i.e. it is no longer found only in specialist clinical genetics services, but is available in dermatology, ENT, cardiology, paediatrics, obstetrics, oncology plus many other specialties (Kotze *et al.* 2015; Brown and Meloche 2016). Given that the results of a genomic test may not only be relevant to the individual who was tested, but also every single one of their biological relatives too, this means that the implications of each test reach outside of the clinical encounter and into the family and extended family. Thus, publics who are currently totally disconnected to the clinical setting have a vested interest in the decisions that their relatives make about the donation of ‘family’ DNA. It is therefore time to bring the broader public into dialogue about the donation and sharing of DNA information. Policy in this area has a responsibility to respect societal attitudes and thus there is an urgent need to gather such attitudes. This work needs to be done internationally, across countries and across all sections of society. The ‘Your DNA, Your Say’ study attempts to do this. The relationship between policy and societal attitudes is a dynamic one; policy needs to take account of public attitudes, but also when it is applied, policy may further shape public attitudes.

## Designing a study

The Participant Values Task Team (PVTT) is an active group within the Regulatory and Ethics Working Group of GA4GH (Participant Values Task Team 2017). The PVTT is an international group of researchers (both non-profit and for-profit), policy makers, clinicians and patient representatives. They met via teleconference during 2015–2016 to support the creation of an empirical study to gather attitudes towards genomic data sharing.

Both research and experience tells us that some publics are unaware of what genomics is (Parry and Middleton 2017), nor how they might access genetic testing (Oliveri *et al.* 2016). Drawing on previous experience of delivering public surveys about genomics (Middleton 2015) a film-survey approach (Middleton *et al.* 2014) was utilised for this study, with the aim of exploring attitudes towards genomic data sharing.

### Use of film

Nine films were created that explain what genomic data sharing (see Video 1).

As we know genomics can be seen a ‘difficult’ or ‘complicated’ subject with the potential to alienate, we deliberately gave the films a non-threatening, open tone, using a child to explain what genomic data sharing is. He uses the shelves in his playroom as an easy to understand metaphor for databases accessed via the Internet. The films are playful and ‘mainstream’ and provide enough information for participants in the survey to be able to answer the questions. As a testament to their appeal across cultures and countries, the films have been awarded official selection status at three mainstream film festivals (in Los Angeles, New York and Bergen in Norway). The purpose of the films is to offer information in as neutral a way as possible, so as not to bias participant attitudes. Thus the films also do not make any positive or negative judgement about the value of genomic data sharing; they simply describe the reality of what is happening within the data sharing arena. A secondary aim of the films was to offer standalone information that could be used by GA4GH partners in explaining what genomic data sharing was. Whilst the GA4GH follows the principle that genomic data sharing is a positive endeavour; the PVTT did not pre-suppose that the public would agree with this position and thus careful consideration was given to the neutral framing and delivery of this project.

### Online survey

**Figure 1.**
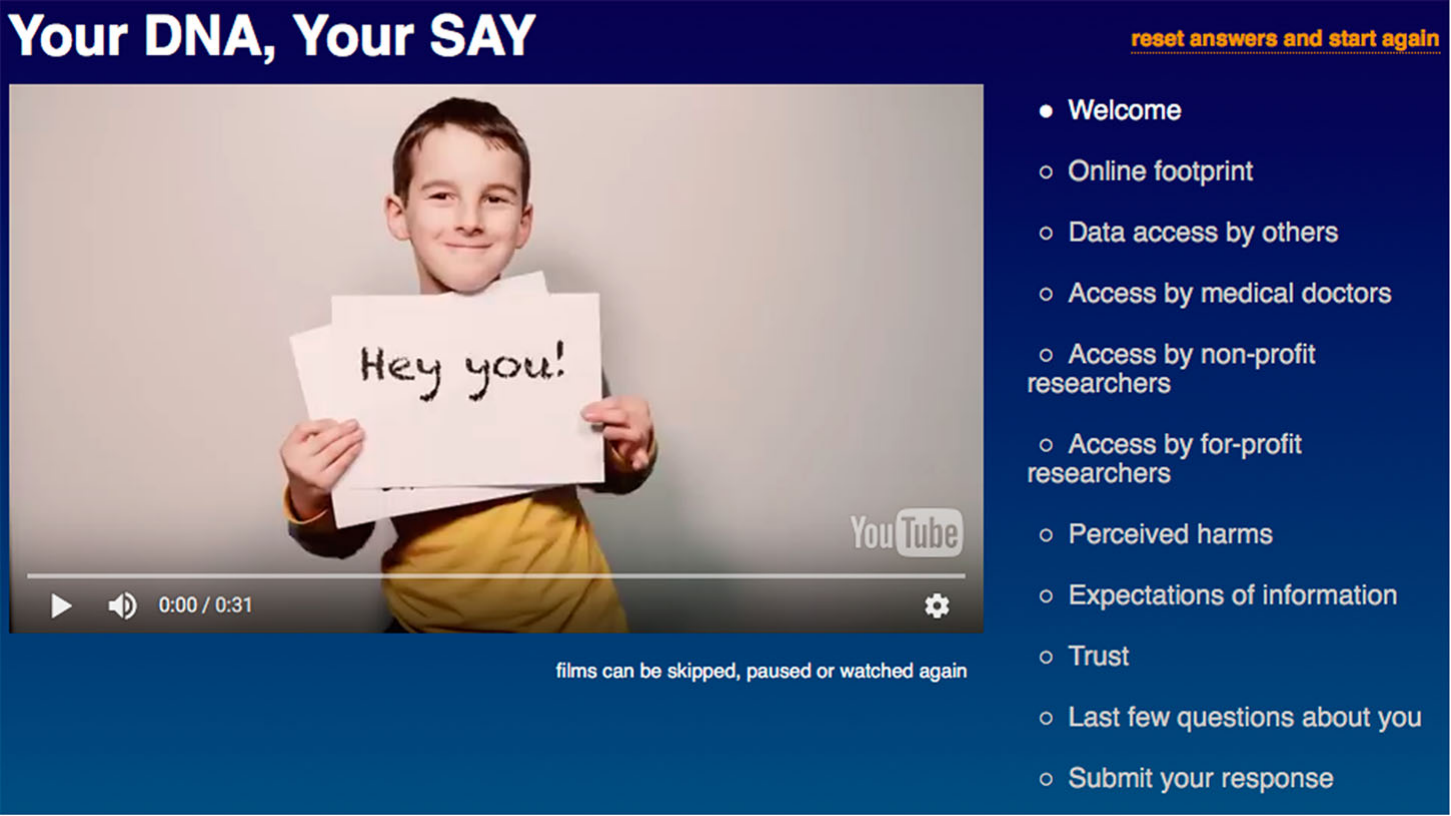
Your DNA, Your Say survey landing page.

The survey has nine sections to it, see Figure 1 for screenshot of survey landing page.

The film and survey begin by orientating the user with the reality of our existing online footprint — most of us have personal data online, whether that be online banking details, social media or file sharing sites for family photos. By framing the context in this way we are asking the user to think about their online presence, so that when we ask them to overlay DNA and medical information onto this landscape, does this change anything for them? The films and survey then explain what DNA information is and how, together with medical information, it can be used to answer questions about health and disease. The subsequent questions explore views on the donation of DNA and medical information for use by clinicians, non-profit and for-profit researchers. The survey explores whether users perceive DNA data to be special in any way (and thus required to be handled differently to other sorts of data) and assesses whether users perceive any harms from the sharing of their data. The survey ends with questions about what trust looks like to participants.

The Your DNA, Your Say survey can be found at: www.YourDNAYourSay.org. It has already been translated into Russian, Polish, French, German and Portuguese, with other languages planned. Recruitment for the English-language version started in 2016 and non-English versions will begin recruitment in 2017.

## Conclusion

Genomic medicine can only be successfully integrated into healthcare if there are aligned programmes of engagement that enlighten the public to what genomics is and what it can offer. Understanding what people want can only happen if you ask them directly (and then do not ignore what they say). The Your DNA, Your Say survey focusses on gathering international attitudes towards the activity of genomic data sharing; these will feed directly into policy that the Global Alliance for Genomics and Health write.

## Supplementary Material

One of the nine films in the survey

## Related video

A video linked to this paper can be found at: http://www.healthcarevalues.ox.ac.uk/anna-middleton-data-sharing-and-participation
